# Effect of fruit extract of *Fragaria vesca* L. on experimentally induced inflammatory bowel disease in albino rats

**DOI:** 10.4103/0253-7613.75660

**Published:** 2011-02

**Authors:** Lalit Kanodia, Mondita Borgohain, Swranamoni Das

**Affiliations:** Department of Pharmacology, Assam Medical College, Dibrugarh, India; 1 Department of Pathology, Assam Medical College, Dibrugarh, India

**Keywords:** Colitis, antioxidant, *Fragaria vesca*, transrectal

## Abstract

**Aim:**

Ulcerative colitis and Crohn’s disease are chronic recurrent inflammatory bowel disease (IBD) of unknown origin. Oxidative stress is believed to be a key factor in the pathogenesis and perpetuation of the mucosal damage in IBD.

**Materials and Methods:**

Ethanolic extract of *Fragaria vesca* (EFFV) fruits was prepared by percolation method and subjected to oral toxicity testing using OECD guidelines. Albino rats were pretreated orally for 5 days with 3% gum acacia in control, EFFV 500 mg/kg in test and 5-aminosalisylic acid (5-ASA) 100 mg/kg in standard groups. Colitis was induced by transrectal administration of 4% acetic acid on 5^th^ day. All the animals were sacrificed with ether overdose 48 hours after colitis induction, and 10 cm colon segment was resected from proximal end. Colon was weighed (for disease activity index) and scored macroscopically and microscopically after histological staining. Biochemical assessments included myeloperoxidase (MPO) and tissue catalase (CAT), glutathione (GSH) and superoxide dismutase (SOD) measurements. Statistical analysis was done using one-way analysis of variance (ANOVA) followed by Dunnett’s “*t*” test.

**Results:**

EFFV showed significant (*P* < 0.05) prevention of increase in colon weight and disease activity index along with decrease in macroscopic and microscopic lesion score as compared to control group. Significant improvement was observed in the levels of MPO, CAT and SOD, except GSH (*P* < 0.05). However, the effect of EFFV was significantly less than 5-ASA (*P* < 0.05).

**Conclusions:**

EFFV at 500 mg/kg showed significant amelioration of experimentally induced IBD, which may be attributed to its antioxidant and anti-inflammatory properties.

## Introduction

Ulcerative colitis (UC) and Crohn’s disease (CD) are collectively known as inflammatory bowel disease (IBD). Although the pathophysiology of IBD is not known with certainty, immunological processes and reactive oxygen species (ROS) have been proposed to contribute considerably to the development of tissue injury.[1] It is thought that some of the intestinal and/or colonic injury and dysfunction observed in IBD is due to elaboration of these reactive species.[2] Commonly used drugs, in particular sulfasalazine and its active moiety 5-aminosalicylic acid (5-ASA), are potent ROS scavengers.[3] In many studies, it has been reported that antioxidants show beneficial effects in experimental colitis.[4] *Fragaria vesca* (wild strawberry) belongs to the family rosaceae. Plants contain flavonoids, tannins, volatile oils, methyl salisylate and borneol.[5] The fruits contain salicylic acid and are beneficial in the treatment of liver and kidney complaints, as well as in the treatment of rheumatism and gout.[6] Acetic acid induced colitis model is similar to human ulcerative colitis in terms of histological features, and has been used extensively in many experimental studies of IBD.[4,7] The effect of various herbal drugs (but not *F. vesca*) on experimental models of IBD has been reported earlier with the antioxidant potential as the main mechanism of action against IBD.[8,9] As the plant *F. vesca* is thought to possess anti-inflammatory and antioxidant properties,[6,10] this study was undertaken to study the effect of *F. vesca* in experimentally induced IBD and to find its probable mechanism of action including its antioxidant potential.

## Materials and Methods

Fresh plants of *F. vesca* were collected from Assam Medical College Campus, Dibrugarh, in the month of March–April 2008. Plant samples were identified and confirmed by Mr. L. Saikia, Reader, Department of Life Sciences, Dibrugarh University (Voucher no. DU/LS/213). The fruits were separated and air dried, which were then crushed and powdered. Ethanolic extract was prepared by percolation method[11] with 95% ethanol, followed by steam evaporation. Exactly 250 g of dry powder was percolated to get a net yield of 30.6 g of concentrated extract (12.24%). All the animals used in the study were taken care of under ethical consideration as per CPCSEA guidelines. The study was conducted after getting approval from Institutional Animal Ethical Committee, Assam Medical College, Dibrugarh (Registration no.-634/02/a/CPCSEA).

Acute oral toxicity was studied with oral administration of extract, using the OECD 2006 guidelines.[12] As per the limit test, female Wistar albino rats were fasted overnight and given 2000 mg/kg of *F. vesca* extract orally, the next day. Animals were observed for 48 hours, with special attention during the first 4 hours, and daily thereafter for a period of 14 days, for any signs of toxicity or mortality. Likewise, five animals were dosed and observed one followed by other. An arbitrary dose of 500 mg/kg was selected for the study, as the extract was found safe even at doses more than 2000 mg/kg without any sign of toxicity or mortality.

Twenty-four healthy Wistar albino rats weighing 150-200 g were divided into four groups with six animals in each group as follows:

Group A (normal control) – received 3% gum acacia 10 mL/kg/day, p.o.
Group B (experimental control) – received 3% gum acacia 10 mL/kg/day, p.o.
Group C (test) – received *F. vesca* extract 500 mg/kg/day p.o.
Group D (standard) – received 5-ASA 100 mg/kg/day p.o.

The animals were pretreated with the respective drugs (volume of drugs was kept constant at 10 mL/kg) for 5 days, along with the normal diet. On the 5^th^ day, animals were kept fasting for 12 hours (overnight) and IBD was induced next morning in Groups B, C and D by administration of 1 mL of 4% acetic acid solution transrectally (TR). Group A (normal control) animals received 0.9% normal saline (TR) instead.[9]

For induction of IBD, an 8-mm soft pediatric catheter was advanced 6 cm from the anus under low-dose ether anesthesia. Rats were in Trendelenburg position during this process and 1 mL of 4% acid or 0.9% normal saline solution was slowly administered TR. The rats were maintained in head-down position for 30 seconds to prevent a leakage, and the rest of the solution was aspirated. After this process, 2 mL of phosphate buffer solution with pH 7 was administered (TR).[9]

All the animals were sacrificed after 48 hours of IBD induction, by ether overdose. Abdomen was opened and colons were exposed. Distal 8 cm of colon was excised and opened by a longitudinal incision. After washing the mucosa with saline solution, mucosal injury was assessed macroscopically using the scale of Morris *et al*.[13] as follows: no damage (0); localized hyperemia but no ulceration (1); linear ulcer without significant inflammation (2); linear ulcer with significant inflammation at one site (3); two or more sites of ulceration and inflammation (4) and two or more sites of ulceration and inflammation or one major site of inflammation and ulcer extending >1 cm along the length of colon (5). Disease activity index (DAI) was measured as the ratio of colon weight to body weight, which was used as a parameter to assess the degree of tissue edema and reflects the severity of colonic inflammation.[8]

Moreover, a 6–8 mm sample block of the inflamed colonic tissue with full thickness was excised from a region of grossly visible damage for histological analysis. Formalin fixed tissue samples were embedded in paraffin and stained with HandE stain. Colonic tissues were scored for histological damage using the criteria of Wallace and Keenan[14] : 0 = intact tissue with no apparentdamage; 1 = damage limited to surface epithelium; 2 = focal ulceration limited to mucosa; 3 = focal, transmural inflammation and ulceration; 4 = extensive transmural ulceration and inflammation bordered by normal mucosa; 5 = extensive transmural ulceration and inflammation involving the entire section.

After scoring, the colonic tissue samples were homogenized with 10 volumes of ice-cold 0.25 M sucrose and centrifuged at 14,000 rpm to measure the biochemical parameters in the resulting supernatant.[15]

### 

#### Biochemical assessments

*Myeloperoxidase (MPO) activity:* To measure MPO activity, colonic samples were minced on ice and homogenized in 10 ml of ice-cold 50 mM potassium phosphate buffer (pH 6.0) containing 0.5% hexadecyl trimethyl ammonium bromide (HETAB). The homogenates were then sonicated and centrifuged for 20 min at 12,000 g. MPO activity was measured spectrophotometrically as follows: 0.1 mL of supernatant was combined with 2.9 mL of 50 mM phosphate buffer in 0.0005% H_2_O_2_. The change in absorbance was measured spectrophotometrically at 460 nm. One unit of MPO activity is defined as the change in absorbance per minute at room temperature, in the final reaction. MPO activity (U/g) = X/weight of the piece of tissue taken, where X = 10 × change in absorbance per minute/volume of supernatant taken in the final reaction.[16]

#### Assessment of antioxidant status in colonic tissue

glutathione (GSH) level was determined according to method of Beutler (1975).[17] The reaction mixture contained supernatant, phosphate buffer and 5,5’-dithio-bis 2-nitrobenzoic acid (DTNB) in a final volume of 10 mL. A blank was also prepared. The absorbance was immediately read spectrophotometrically at 412 nm before and after addition of DTNB. The values were determined from the standard curve.

Superoxide dismutase (SOD) was measured according to the method of Fridovich (1983).[18] Assay medium consisted of 0.01 M phosphate buffer, 3-cyclohexilamino-1-propanesulfonic acid (CAPS), saturated NaOH with pH 10.2, solution of substrate (0.05 mM xanthine, 0.025 mM *P*-iodonitrotetrazolium violet) and 80 *μ*L xanthine oxidase. Absorbance was read spectrophotometrically at 505 nm. SOD was expressed as U/mg of proteins.

Catalase was measured by the method of Beers and Sizer.[19] Phosphate buffer (2.5 mL, pH 7.8) was added to supernatant and incubated at 25°C for 30 min. After transferring into the cuvette, the absorbance was measured at 240 nm spectrophotometerically. Hydrogen peroxide (650 *μ*L) was added and change in absorbance was measured for 3 min. Values were expressed as *μ*mol/min/mg of protein.

Statistical analyses were done using one-way analysis of variance (ANOVA) followed by Dunnett’s multiple comparison tests. *P* < 0.05 was considered as significant.

## Results

**Figure 1:**
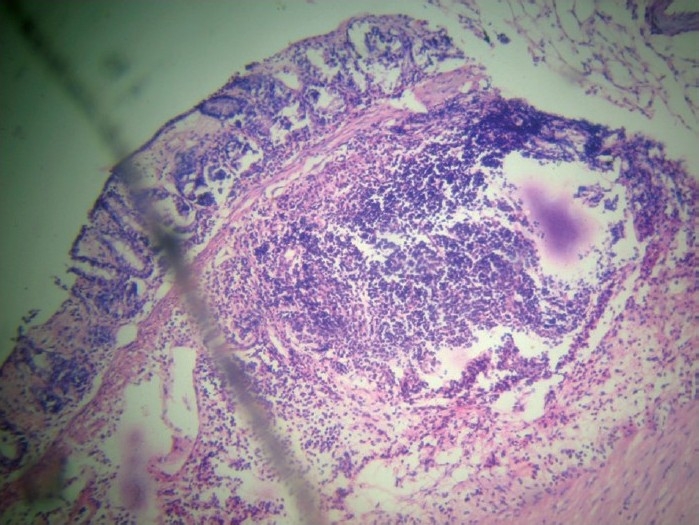
Group B (experimental control): Extensive necrosis with transmural infiltration.

Acetic acid administration to the experimental control group caused significant macroscopic ulcerations and inflammations (*P* < 0.05) in rat colon along with significant mucosal injury [Figure 1] microscopically (*P* < 0.05), when compared to the normal control group (*P* < 0.05). Also, there was significant derangement of biochemical parameters including tissue levels of MPO, GSH, SOD and catalase (*P* < 0.05), showing oxidative stress due to colon damage and colonic inflammation [Table 1].

**Table 1:** Effect of *Fragaria vesca* on experimentally induced IBD

*Groups*		*Macroscopic score*	*Disease activity index (DAI)*	*Microscopic score*	*Tissue CAT (*μ*mol/min/mg of proteins)*	*Tissue GSH (nmol/mg of proteins)*	*Tissue SOD (U/mg of proteins)*	*Tissue MPO (U/g)*
Normal control		0.33 ± 0.21^a^,^d^	0.67 ± 0.042^a^,^d^	0 ± 0^a^,^c^	376.2 ± 6.13^a^,^c^	154.1 ± 5.8^a^,^c^	6.9 ± 0.33^a^,^c^	0.33 ± 0.03^a^,^c^
Experimental control		4.67 ± 0.21^c^	1.14 ± 0.045^c^	4.7 ± 0.21^c^	109.5 ± 5.16^c^	84.6 ± 4.07^c^	3.4 ± 0.24^c^	2.9 ± 0.06^c^
*F. vesca* extract		2.0 ± 0.36^a^,^c^	0.9 ± 0.003^a^,^c^	3.0 ± 0.36^a^,^c^	226.3 ± 7.67^a^,^c^	97.8 ± 5.9^b^,^c^	5.2 ± 0.32^a^,^c^	1.8 ± 0.07^a^,^c^	
5-ASA		1.0 ± 0.26^a^	0.76 ± 0.033^a^	1.5 ± 0.22^a^	304.9 ± 9.23^a^	128.4 ± 6.17^a^	6.4 ± 0.27^a^	0.76 ± 0.033^a^	
ANOVA	F	50.26	28.29	70.37	250.8	31.51	28.44	333.4
	df	20,3	20,3	20,3	20,3	20,3	20,3	20,3	
	*P*	<0.05	<0.05	<0.05	<0.05	<0.05	<0.05	<0.05

Values expressed as mean ± SEM, *n* = 6;

a *P* < 0.05;

b *P* > 0.05 when compared to experimental control;

c *P* < 0.05;

d *P* > 0.05 when compared to standard

**Figure 2:**
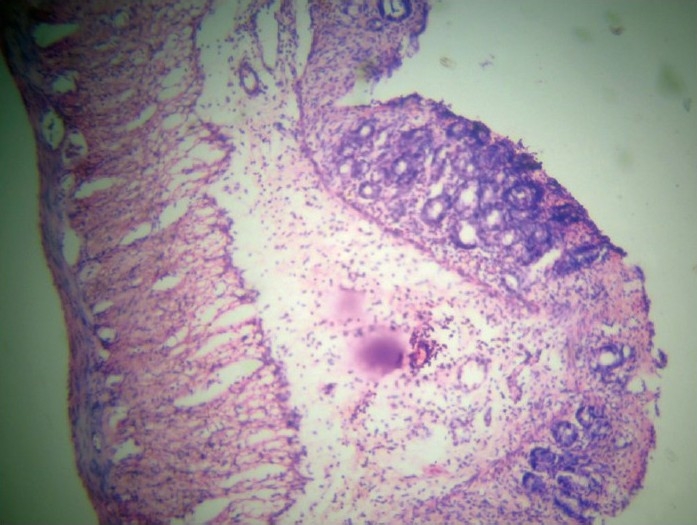
Group C (*F. vesca*): Infiltration up to submucosa, architecture maintained.

Ethanolic extract of *F. vesca* (EEFV) fruits showed significant activity against experimentally induced IBD when compared to the experimental control (*P* < 0.05), with near normalization of colon architecture both macroscopically as well as microscopically [Figure 2]. Tissue oxidative stress was reduced with significant improvement in tissue levels of SOD and CAT (*P* < 0.05), showing its antioxidant potential, although there was no significant difference in GSH levels when the two groups were compared (*P* > 0.05). Also, significant improvement in the levels of MPO was observed (*P* < 0.05) [Table 1].

**Figure 3:**
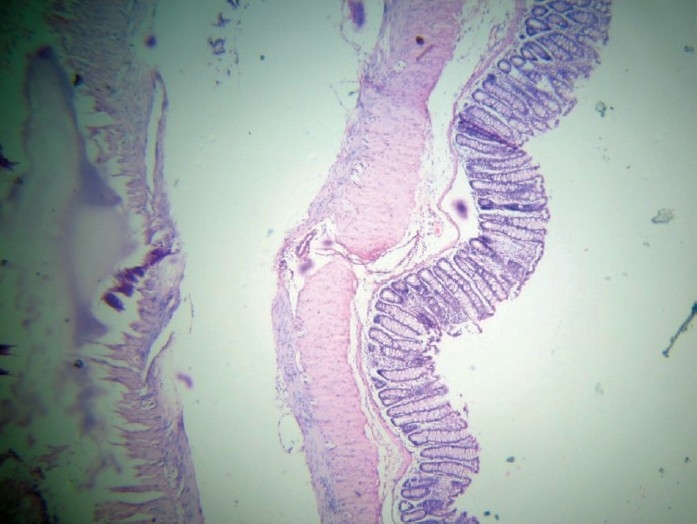
Group A (normal control): Normal mucosal architecture

**Figure 4:**
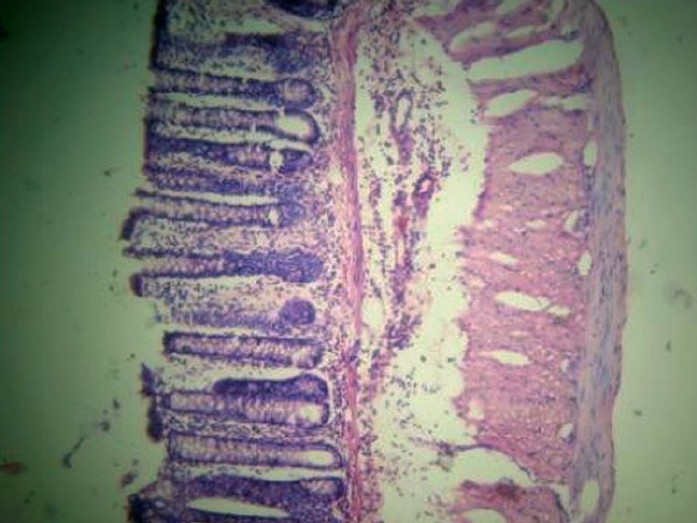
Group D (5-ASA): Near normalization of architecture with mucosal infiltration only.

As for the standard drug 5-ASA, its activity against IBD was significantly better than *F. vesca* extract with regard to all the parameters (*P* < 0.05). When compared to the normal control, 5-ASA showed near normalization of DAI, macroscopic score and microscopically [Figures 3 and 4], as there was no significant difference between the two groups, i.e., the normal control group and 5-ASA group (*P* > 0.05), thus showing its potent activity against experimentally induced IBD [Table 1].

## Discussion

The results of this study have shown that ethanolic extract of fruit extract of *F. vesca* has got a good potential to suppress experimental colitis in rats, as indicated by macroscopic, microscopic and biochemical evaluations. Acetic acid induced colitis model is similar to human ulcerative colitis in terms of histological features. It affects the distal colon portion and induces non-transmural inflammation, massive necrosis of mucosal and submucosal layers, mucosal edema, neutrophil infiltration of the mucosa and submucosal ulceration. The protonated form of the acid liberates protons within the intracellular space and causes a massive intracellular acidification resulting in massive epithelial damage. Inflammation is the pathogenesis of IBD and several pathways are associated with inflammatory response in IBD.[20] The inflammatory response initiated by acetic acid includes activation of cyclooxygenase and lipooxygenase pathways.[21,22]

Fruits of *F. vesca* have been found to contain salicylic acid[6] which is a known anti-inflammatory agent that acts by inhibiting cyclooxygenase enzyme. Therefore, this might be its probable mechanism of anti-inflammatory action. Also, flavonoids found in the *F. vesca* plant[5] possess anti-proliferative activity that causes a decrease in the weight and volume of contents of granuloma in inflammation.[23]

Oxidative stress is believed to play a key role in the pathogenesis of IBD-related intestinal damage.[24] Intestinal mucosal damage in the IBD, including Crohn’s disease and ulcerative colitis, is related to both increased free radical production and a low concentration of endogenous antioxidant defense.[25] As proved by the above study and also as described in literature,[10] the fruit extract of *F. vesca* possesses significant antioxidant property, proving its role in the management of experimentally induced IBD.

Hence, it can be concluded from this study that ethanolic extract of fruits of *F. vesca* has potent activity against experimentally induced IBD, due to its anti-inflammatory and antioxidant properties. Further investigations for its clinical utility are warranted.
